# Factors leading to delayed and challenging presentation of benign breast lumps in young females

**DOI:** 10.12669/pjms.39.1.6647

**Published:** 2023

**Authors:** Saira Saleem, Sundus Tariq, Saba Tariq, Sofia Irfan, Farhan Javed

**Affiliations:** 1 Dr. Saira Saleem, MBBS., FCPS., FRCS. Professor General Surgery, Madinah Teaching Hospital & The University of Faisalabad, Faisalabad, Pakistan; 2 Dr. Sundus Tariq, MBBS., MPhil., PhD. Professor Physiology, University Medical & Dental College, The University of Faisalabad, Faisalabad, Pakistan; 3 Dr. Saba Tariq, MBBS., MPhil., PhD. Professor Pharmacology and Therapeutics, University Medical & Dental College, The University of Faisalabad, Faisalabad, Pakistan; 4 Dr. Sofia Irfan, MBBS., FCPS. Assistant Professor General Surgery, Allied Hospital & Faisalabad Medical University, Faisalabad, Pakistan; 5 Dr. Farhan Javed, Associate Professor General Surgery, Madinah Teaching Hospital & The University of Faisalabad, Faisalabad, Pakistan

**Keywords:** Benign Breast disease, Social Stigma, Social Isolation, Fibroadenoma, Hypertrophy

## Abstract

**Background and Objective:**

A delayed presentation of benign breast diseases may lead to a therapeutic challenge when they enlarge enormously or become multiple. Aim of this study was to evaluate the factors leading to delayed presentation of benign breast lumps.

**Methods:**

This cross-sectional study was conducted at Madinah Teaching Hospital and Allied Hospital, Faisalabad from January 2019 to October 2021. One hundred and forty five female patients were selected by non-probability purposive sampling. Patients with large size (>5cm) or multiple benign breast lumps were included. An interview was conducted using structured questionnaire translated in Urdu. Factors leading to delayed presentation and social impact scale for stigma were evaluated.

**Results:**

Patients had a mean age of 26.52 ± 6.90 years. The average delay of seeking medical care was 8.48 ± 8.41 months. Factors leading to delayed presentation were; lack of knowledge n=112 (77.2%) and parda (religious issues) n=112 (77.2%), followed by poverty n=109 (75.2%), and fear of cancer n=90 (62.1%). All the sub-scales of stigma, i.e., social rejection, financial insecurity, internalized shame and social isolation were high in late presenters, though, only financial insecurity was significantly high in late presenters (p=0.03).

**Conclusion:**

Lack of awareness, socioeconomic factors and disease related stigma were the main factors related to delayed presentation in young females with benign breast diseases. Addressing these factors may improve timely diagnosis and management of delayed and challenging cases.

## INTRODUCTION

Benign Breast Disease (BBDs) are the most common cause of breast problems in females and are more prevalent than the malignant breast diseases.^1^,^2^ Fibroadenomas are the most common benign tumors of the breast under the age of 30 years with a peak age-incidence around 17–20 years.^1^ Occasionally, they grow to a bigger size resulting in a Giant Fibroadenoma (GFA). Giant fibroadenomas are larger than 5cm and are the most common cause of unilateral breast enlargement.^3^,^4^ Cystosarcoma phyllodes is also one of the causes of large size breast masses in younger patients.^1^ Occasionally we find cases of atypical hyperplasia in patients with benign breast lumps, that carries a risk of malignancy. Early diagnosis and treatment as well as awareness regarding the risk of breast cancer is very important in these patients.^2^

A delay in seeking medical care in BBDs is related to progression of tumour to large size and multiplicity that pose a diagnostic and therapeutic challenge. Simple surgical excision in larger and multiple lesions result in a displeasing, loose, ptotic breast and may require secondary surgery or even removal of entire breast.^5^ Thus surgeon has to face a reconstructive challenge similar to when a large malignant neoplasm is removed.^6^ Recently, with the invent of oncoplastic breast surgery, breast conservation with good aesthetic results are seen but this needs specialized skills and training.^7^

Young females with breast lumps have much emotional distress and feel shame of telling even their mothers and then mothers take it as social stigma and don’t seek timely medical care.^8^ Overall burden of illness and complexity of management is increased in patients with health related stigma. Social or public stigma represents the central stigma type and refers to the stigmatizing responses of others towards the patient.^9^ Although importance of recognizing and exploring cancer related stigma is gaining attention,^10^ stigma related to benign breast diseases is not addressed in literature that needs to be focused to avoid its negative impact on population health. In this study, we explored the factors related to late presentation of females with benign breast disorders that may be large sized, multiple or associated with enormous hypertrophy and asymmetry. Addressing these factors could improve disease prognosis and outcome.

## METHODS

This cross-sectional study was conducted at Breast Surgery Department of Madinah Teaching Hospital and Allied Hospital, Faisalabad from January 2019 to October 2021.

A sample size of 150 was calculated using the formula, n = (Z_α/2_+Z_β_)^2^ * (p_1_(1-p_1_)+p_2_(1-p_2_)) / (p_1_-p_2_)^2^ where Z_α/2_ is the critical value of the normal distribution at α/2 (for a confidence level of 95%, α is 0.05 and the critical value is 1.96), Z_β_ is the critical value of the normal distribution at β (for a power of 80%, β is 0.2 and the critical value is 0.84) and p_1_ and p_2_ are the expected sample proportions of the two groups (42% and 58%) taken from the literature.^11^ After review five subjects were excluded from the study during analysis as they were not meeting the inclusion criteria. Total sample size for the study was 145.

### Ethical Approval:

Study was approved by the Research and Ethical Committee of The University of Faisalabad (Ref # TUF/Dean/2018/29) dated December 3, 2018. A written informed consent was taken from all the participants about the publication of data for research and educational purpose.

**Fig.1:**
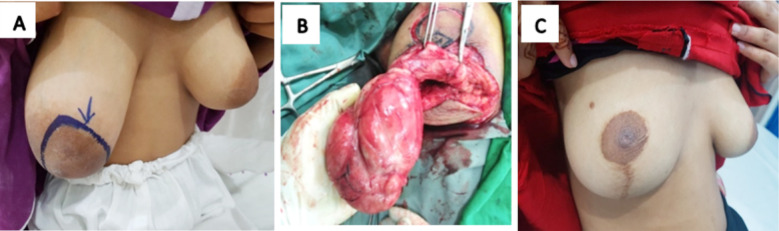
An adolescent girl with giant fibroadenoma and unilateral breast hypertrophy. A: Preoperative B: Resection of lump and reduction mammoplasty, C: Post-operative outlook after oncoplastic breast surgery.

Study population was recruited using non-probability, purposive sampling technique. Patients with benign breast lumps who had a delay in presentation with a lump of larger size (>5cm) or multiple numbers or associated hypertrophy and challenging presentation were included (Fig 1a,b,c). Benign breast lumps were diagnosed on clinical assessment and breast ultrasound followed by FNAC. Patients of 12 to 40 years age who were physically active were included in the study. Factors causing delayed presentation in young patients with benign breast diseases were evaluated. Delay was defined as “More than three months interval between the appearance of first symptom to consultation from doctor”.^12^ Patients with breast cancer, those who presented earlier than three months and the females who did not remember the approximate date of appearance of lump were excluded. Also, physically inactive or handicapped patients were excluded to avoid the confounding factor in delayed presentation.

Patients who presented with a delay (more than three months interval between the appearance of first symptom to consultation from doctor) were divided into two groups; early and late presenters. Early presenters group included those females who presented between three to six months and late presenters included females who presented after six months of appearance of breast lump.

All participants were examined by a breast surgeon and those fulfilling the inclusion criteria were interviewed by expert researchers at breast clinic in a quiet and private place. A questionnaire was designed including the sociodemographic questions and social impact scale (SIS).^13^ The national language of Pakistan is Urdu so qualified translators available in The University of Faisalabad translated the questionnaire from English to Urdu. A second translator did a back translation from Urdu to English. Differences in the translation were identified, adjusted and another back translation was made. A pilot study was conducted first to establish the validity and reliability of the instrument. The questionnaire was validated and the Cronbach’s alpha showing the reliability of the data was 0.88. The interviews were conducted in Urdu using the designed questionnaire. All the participants were able to understand and respond in Urdu.

Sociodemographic characteristics and information about date and type of initial symptom of breast disease, family history of breast cancer, marital status, number of children, menstrual history and social support were also obtained. Disease related stigma was measured using social impact scale (SIS),^13^ The SIS is composed of 24 items and divided into four domains: social isolation, internalized shame, social rejection and financial insecurity. The responses were recorded on a four points Likert scale where one represented strongly disagree, two disagree, three agree and four represented strongly agree.

Data analysis was performed using statistical package for social sciences (SPSS version 26). Percentages and proportions were given for qualitative variables while mean ± SD was given for quantitative variables. Percentages were compared using chi-square and fisher exact test while means were compared using independent sample t-test. The effect of independent variable on the predictive variable was seen using ordinary least square regression analysis. A p-value less than <0.05 was considered significant.

## RESULTS

The study included 145 females with large or multiple benign breast lumps. Giant fibroadenoma was the most common histopathological finding (59.3%), followed by phyllodes tumour (6.9%) and fibrocystic disease (6.9%). Some of the patients with delayed presentation had marked hypertrophy and asymmetry of the breast.

The mean age of the study population was 26.52 ± 6.90 years. The average time of consultation after the appearance of benign lump was 8.48 ± 8.14 months. The general demographic features, important features in history and level of independence of the females is presented in Table-I.

**Table-I:** General demographic features of study population.

Variables		Young females (n = 145)
Age (years)		26.52 ± 6.90
Age of menarche (years)		12.50 ± 0.60
Time of consultation (months)		8.48 ± 8.14
Time of surgery (months)		10.58 ± 9.30
Marital status	Unmarried	71 (49.0)
	Married	74 (51.0)
Residence	Rural	74 (51.0)
	Urban	71 (49.0)
Socioeconomic status	< 50,000 pkr/month	141 (97.2)
	≥ 50,000 pkr/month	4 (2.8)
Education	Primary	73 (50.3)
	Secondary	61 (42.1)
	Graduate	11 (7.6)
Occupation	Housewife	116 (80.0)
	Working	20 (13.8)
	Student	9 (6.2)
Smoking	Yes	2 (1.4)
	No	143 (98.6)
Menstrual history	Regular	126 (86.9)
	Irregular	19 (13.1)
Family history of this disease	Yes	10 (6.9)
	No	135 (93.1)
Family history of any breast lesion	Yes	14 (9.7)
	No	131 (90.3)
Use of hormonal contraceptives	Yes	11 (7.6)
	No	134 (92.4)
Level of Independence	Self-sufficient	65 (44.8)
	Help from family	72 (49.7)
	Help from friends	4 (2.8)
	Receive home health care	4 (2.8)

Values are presented as mean ± SD and n (%).

The most common patient perspective for delayed presentation was lack of knowledge n=112 (77.2%) and parda (religious issues) n=112 (77.2%), followed by poverty n=109 (75.2%), fear of cancer n=90 (62.1%), non-availability of healthcare services n=86 (59.3%) and availing alternative treatment options n=66 (45.5%).

The comparison of anthropometrics and four sub-scales of social impact scale with early (<6 months) and late presenters (>6 months) is shown in Table-II. All the sub-scales of stigma, i.e., social rejection, financial insecurity, internalized shame and social isolation were high in late presenters as compared to early presenters though, only financial insecurity was significantly high in late presenters (p=0.030).

**Table-II:** Comparison of anthropometrics and social impact scale (SIS) scores between two groups of late presenters.

	<6 months n=50 Mean ± SD	≥6 months n=95 Mean ± SD	p-value
Age (years)	26.58 ± 6.85	26.49 ± 6.96	0.944
Height (meters)	1.59 ± 0.05	1.59 ± 0.06	0.312
Weight (kilograms)	56.94 ± 6.06	58.53 ± 9.09	0.269
BMI	22.38 ± 2.21	23.41 ± 4.69	0.146
Menarche age (years)	12.51 ± 0.50	12.50 ± 0.66	0.925
Social Rejection	21.18 ± 3.31	22.07 ± 3.50	0.139
Financial Insecurity	7.28 ± 1.07	7.72 ± 1.17	0.030*
Internalized shame	15.12 ± 2.44	15.23 ± 2.84	0.814
Social Isolation	19.90 ± 3.27	20.04 ± 3.17	0.800

Significant association of early and late presenters were also seen with the educational level of the study subjects, as less educated females had more delay, Fisher = 6.08, *p*-value = 0.04.

The impact of various variables on the four subscales of stigma (social impact scale) using ordinary least square regression analysis is shown in Table-III.

**Table-III:** Impact of various variables on social impact scale using ordinary least square regression analysis.

	Social Rejection	Financial Insecurity	Internalized shame	Social Isolation

	B	B	B	B
Age	-0.052	-0.002	-0.169	-0.079
Weight	-0.825	-0.289	0.081	0.895
Height	0.485	0.208	-0.015	0.493
BMI	1.024	0.324	-0.279	0.830
Monthly income	0.121	-0.010	0.076	0.099
Education	-0.159	-0.182	-0.057	-0.140
Occupation	0.039	0.064	0.186*	0.035
Level of Independence	-0.043	-0.127	-0.257*	-0.274*
Smoking	0.091	0.187	-0.024	0.042
Family History of this diseases	0.063	0.180	0.106	0.125
Family History of Breast Cancer	0.019	-0.151	-0.104	-0.106
Use of Hormonal contraceptives	-0.072	0.108	0.155	0.071
Fear of Cancer	0.233*	0.128	0.067	0.133
Limited health facilities	-0.018	-0.009	-0.055	0.101
Lack of Knowledge	0.106	0.080	-0.110	0.051
Alternative treatment	0.032	0.022	0.005	-0.010
Parda & Religious issues	-0.021	0.018	0.076	0.087
Poverty	0.084	-0.024	0.104	0.211*
R square	0.185	0.136	0.219	0.245

B is standardized coefficient beta,

* =p< 0.05 is statistically significant.

The significant positive predictor of social rejection was fear of cancer. The significant negative predictor of internalized shame was level of independence while the significant positive predictor was occupation. The significant negative predictor of social isolation was level of independence while its significant positive predictor was poverty.

## DISCUSSION

In the past, most of the research was focused on addressing the factors that cause late presentation of breast cancer.^12^,^14^,^15^ However, information and research on late presentation of BBDs is lacking. Delayed presentation of patients with large or multiple benign lumps poses a surgical challenge and in certain cases increases the risk of malignancy. In this study we explored the factors leading to delayed presentation of benign breast disorders.

Most common benign breast disease found in our study was the giant fibroadenoma (59.3%). In many cases of giant fibroadenoma there was associated breast hypertrophy and asymmetry of two sides. So, we had to face a reconstructive challenge similar to when a large malignant breast neoplasm is removed as described by Hiller and colleagues. Removing such bigger lumps by simple surgical techniques may lead to poor cosmetic results.^6^ We found few cases of atypical ductal hyperplasia (3.4%) that increase the risk of malignancy by four to tenfold if not diagnosed and treated timely.^14^ In our study nine patients (6.2%) had multiple fibroadenomas. Worsham MJ et al. and colleagues showed an association of multiplicity of benign lumps with subsequent risk of malignancy.^16^

In our patients a mean delay of 8.48±8.14 months was observed. Out of which 65.51% had a delay of > 6 months. The most common patient perspective for delayed presentation was lack of knowledge and parda. Similarly, a study conducted at Islamabad for breast cancer patients, showed main reasons for delay to be lack of knowledge, lack of availability of health care services and religious reasons.^11^ A recent study conducted at PINUM, Faisalabad described lack of knowledge and disease awareness to be the most common factors in our community as well as in other Muslim countries.^17^

Majority of our patients belonged to lower socioeconomic status and due to lack of financial resources they were unable to bear health care expenditures. Thus, poverty posed another barrier for timely consultation for treatment as described by 75.2% of our patients. This is similar to the approach of patients with breast cancer as described in PINUM study.^17^ Educational status also influenced delay in presentation and less educated females had more delay as compared to educated females. Similar influence was presented in an Iranian study.^12^ Manzoor S et al. also highlighted importance of awareness and patient education to improve the delayed presentation.^18^

We observed that benign breast diseases are also perceived as stigma and cause negative connotations and delay in seeking appropriate medical care leading to their progression. Young unmarried females considered it difficult to find husband and getting married, if the disease is disclosed. Married females had other issues like those living in a joint family system, described that they will face social isolation, social rejection or internalized shame to discuss their disease with other family members. In our study, all the sub-scales of stigma, i.e., social rejection, financial insecurity, internalized shame and social isolation were high in late presenters, financial insecurity, however had a significant association with delayed presentation. A Chinese study also showed that the subscale of stigma (SIS), financial insecurity has the highest average score in breast cancer survivors.^19^

Regarding influence of sociodemographic factors on perceived stigma, we found females with less income levels and those with fear of cancer were more stigmatized, while independent and working females were less stigmatized. Iddrisu from Ghana described that lack of financial independence contributed to stigma towards breast cancer and it does not allow patients to make decisions for early diagnosis and treatment. Such females feel more social isolation and experienced withdrawal and strange reaction from society, so they don’t disclose their diagnosis even to friends and family members.^8^ We also found that stigma is related to educational level of the patients as females with less educational level were more stigmatized, while more educated and working females felt less stigmatized. Holman D et al. also described that woman with breast cancer who had not received any formal education perceived high levels of stigma.^20^ Better level of education is probably related to better health awareness and less stigma.^21^

Although factors like, age, marital status and disease history, are not modifiable, socio-cultural variables such as people’s knowledge and assumptions about breast disease, its signs and symptoms, and the perceived stigma, are adaptable as described by Gulzar F et al.^14^ Similarly, if we identify and appropriately address the factors leading to a delayed presentation in benign breast disorders, we can improve the management and outcome of these cases. As there is no previous study, exploring the impact of factors causing delayed presentation of benign breast d

### Limitations of the study:

Small sample size was the main limitation of this study.

## CONCLUSION

Lack of knowledge, low socioeconomic status and social stigma related to breast disease were the main factors that caused delay and posed a barrier to seek medical care in young females with benign breast diseases. Social stigma in these females is significantly associated with poverty, less educational level, lack of independence and fear of cancer. A delayed presentation poses a therapeutic challenge. Our study detected important barriers of health seeking in such patients and thus provides an insight for developing effective strategies to timely manage such issues in young females.
